# American Dental Society of Europe

**Published:** 1888-03

**Authors:** E. A. Galbreath

**Affiliations:** Hannover


Adjourned.

---

### AMERICAN DENTAL SOCIETY OF EUROPE.

### FIFTEENTH ANNUAL MEETING AT COBLENTZ, GERMANY, SEPTEMBER, 1887.

Reported for the Independent Practitioner by Dr. E. A. Galbreath, Hannover.

Concluded From Page 91.

### THURSDAY AFTERNOON SESSION CONTINUED.

The President announced a paper by Dr. Miller; subject "The Effect of Food upon the Teeth."

*Dr. Miller.*—It is not my object to enter into a detailed account of the experiments I have been making on this subject, as they are not yet, by any means, completed. I have found the difficulties vastly greater than I anticipated, and instead of doing the whole work in six to eight months, as I thought I could in the beginning, it will require at least three or four years. The object of my experiments, as I stated at our last meeting, is to determine whether or not the teeth, either during their development or at any subsequent period, may be influenced by the character of the food given; in particular, by an abundance or by a lack of lime-salts. This I consider to be a question which can be settled by experiment alone, individual opinions being, as then stated, very nearly worthless. It is true that the majority of experienced dentists with whom I have spoken on the subject have answered my question in a positive sense, without, however, being able to give any conclusive evidence; others are of a directly opposite opinion, and for no better reasons. I take this opportunity to state that my object is not to support any particular view, but to obtain results upon which an opinion may be scientifically based. I showed in my paper last year, which was simply an introduction to the subject, that under certain specified conditions the amount of lime-salts taken by children is less than that necessary to properly build up the osseous system. I also made a number of experiments on grown dogs, obtaining results of which I said: "Although on the whole it might appear that a change had been produced in the proportion of lime-salts to organic matter in the teeth, yet the number of experiments is too small and the results not sufficiently positive to admit of drawing any definite conclusions." The annotation on the above communication in the *London Lancet,* as well as the remarks on the same by Messrs. Sewell and Ch. Tomes, produced an incorrect impression of the contents of my article, Sewell even going so far as to make a rather lengthy quotation, which he says is evidently from Miller, but which in fact was not at all from Miller.

During the last twelve months my experiments have been made exclusively on young animals (mice and dogs). Attempts at experiments on cats failed through the premature death of the animals. In each series of experiments the litter of pups or mice was separated into two groups, one of which was fed normally, the other on artificial milk (containing all the constituents of milk except lime), sugar, fat and peptones. You will all be convinced as to the effects of such a regimen upon the bones, as you see the epiphyses are exactly like little sponges, and the shafts can be tied into knots. As for the teeth, I have been very much surprised to find how very little they seem to have been affected, both as to time of eruption and the development of the roots. What the effect may have been upon the chemical composition I cannot state, as the analyses have not yet been made, nor do I wish to enter into a discussion of the matter until my experiments have been completed. My results, so far, I may say, appear to be markedly different from those of Galippe, who is of the opinion that the teeth are the first to be affected. I found the bones decidedly more altered, at least macroscopically, than the teeth.

Dr. Miller also gave a resumé of the most important papers which have been produced during the year on histological and pathological dental subjects, dwelling at length on the recent communications of Bödecker and Heitzmann in the INDEPENDENT PRACTITIONER, relative to the development of the teeth. The views of these authors, as well as the accepted theories, were illustrated by large drawings made by Dr. C. H. Abbot. Dr. Miller represented Drs. Bödecker and Heitzmann as at present standing alone in their views, and as being opposed by some of the best dental pathologists in America, who declared themselves unable to see, even in preparations made by Drs. Bödecker and Heitzmann, what these histologists claim to see, and what Dr. Heitzmann has made so plain in his drawings.

Many other histologists are at present testing the views of Drs. Bödecker and Heitzmann, and in a few months we may hope to hear a conclusive substantiation or refutation of them.

The Society then going into executive session, the following resolutions were offered:

*Resolved,* That the constitution be so changed as to permit of the next meeting being held two years hence, in 1889, in Paris, the date to be announced by the executive committee.

*Resolved,* That five hundred marks a year for the next two years be set aside for microscopical researches.

*Resolved,* That the Society, recognizing the professional devotion and generosity of Dr. Herbst, begs to thank him for his kindness in sending for exhibition specimens illustrating several of his methods of operating.

*Resolved,* That a committee, consisting of Drs. Jenkins and Crane, draft resolutions expressive of the feelings of the members upon the death of Dr. Abbot.

The balloting for officers gave the following result:

*President*—Dr. Elliot.

*Vice-President*—Dr. Sachs.

*Treasurer*—Dr. Bryan.

*Secretary*—Dr. Patton.

Drs. Jenkins and Crane here presented the following report, which was adopted and ordered inserted in the minutes :

WHEREAS, It has pleased the Divine Providence to remove by death our beloved friend and colleague, Dr. F. P. Abbot, therefore,

*Resolved,* That by this event we have lost one who was not only one of the brightest ornaments of our profession, but who had especially endeared himself to the members of this Society by the amiability of his character, and won their highest admiration by the brilliant qualities of his mind.

In presenting this report, Dr. Jenkins said :

The friend whose loss we all so deeply mourn has stood for more than thirty years at the very head of our profession in Germany. He found it a little respected mechanical art ; he left it a recognized and honored profession. The position which American dentistry occupies to-day is due, in a degree which none of us can estimate, to his courage, his great skill as an operator, the versatility of his mind, the tenderness of his heart, and his profound conscientiousness. Since early childhood he had been the victim of severe asthmatic attacks. What would have been with a man of less determined spirit an excuse for idleness, was to him but a spur to greater industry and an occasion of active improvement of the intervals of suffering. The achievements of his life would put us unhampered ones to shame, did we not know that he was born a hero. His malady never brought impatient or complaining words to his lips. He saw in middle life his entire fortune swept away by a single blow, and with unimpaired cheerfulness and dauntless courage began life anew the next morning. The friends who had come to speak words of sympathy to him in affliction presently found themselves led to confide to his tender and unselfish heart their personal sorrows, in consoling which he seemed to forget his own. No words can do justice to his charities. I speak not only of his princely charities of the purse, but of that nobler charity of the heart, by means of which he has raised so many of us to higher thought and better living. He was a patriot, and in the dark days of the civil war his house was the rallying point of those who refused to despair of the Republic. He was a Christian, and his life was fragrant with thoughts and words and deeds of piety and benevolence, as he taught by precept and example the fatherhood of God and the brotherhood of all mankind. I love to think of our departed brother in the words with which Tennyson has painted his ideal knight:—

> "Who revered his conscience as his king,
> Whose glory was redressing human wrong.
> Who spoke no slander; no, nor listened to it.
> Who loved one only, and who clave to her;
> O, selfless man, and stainless gentleman."

The President appointed Dr. Miller to conduct the President-elect to the chair.

President Elliot appointed as the Executive Committee, Dr. Crane, Dr. Miller, and by general request Dr. Elliot.

The President appointed as the Membership Committee, Dr. Sachs, Dr. Jenkins, Dr. George.

As Committee on Dental Education, Dr. Miller, Dr. Patton, and upon motion Dr. Elliot.

The Society then adjourned.

---

### CENTRAL DENTAL ASSOCIATION OF NORTHERN NEW JERSEY.

REPORTED FOR THE INDEPENDENT PRACTITIONER.

